# 

**Published:** 1899-07

**Authors:** 


### CONTENTS.

COMMUNICATIONS.

Educational Methods ........ 301

PROCEEDINGS.

Southern Branch, National Dental Association, Second Annual Meeting, New Orleans, La., February 9, 10, 11 and 13, 1899 ...... 313
Microscopic Effects of Cataphoresis ........ 313
Oral Hygiene ........ 314
Sectional Bridgework ........ 315
Plain Rubber Teeth in Bridgework ........ 316

SELECTIONS.

Mouth Breathing ........ 319
Modern Parisian Practice—The French Therapeutics of the Period 322
Ancient and Modern ........ 325
Durability of Osteo Fillings ........ 329
Foreign Body Imbedded in the Cheek for Eight Months ........ 330
Nerve Transplantation ........ 331
A Note on a Case of Swallowing False Teeth ........ 332
Setting of Plaster of Paris ........ 333
An Inquest on the body of Harry Westley Boyle Haines ........ 334

COMMENCEMENTS.

New York Dental School ........ 334
The Columbia University, Washington, D. C., Dental Department... 335
Medico-Chirurgical College of Philadelphia, Department of Dentistry 335
Colorado College of Dental Surgery ........ 335
Dental College of the Province of Quebec ........ 336
Ohio College of Dental Surgery ........ 336
University of Michigan—Dental Department ........ 337
Missouri Dental College ........ 337
Western Reserve University Dental College ........ 338
College of Physicians and Surgeons of San Francisco ........ 338
Indiana Dental College ........ 339
Ohio Medical University—Department of Dentistry ........ 339
Miami Dental College ........ 340
Keokuk Dental College ........ 340

NOTICES.

National Dental Association Meeting ........ 340
Twenty-ninth Annual Session of the New Jersey State Dental Society ........ 345

EDITORIAL.

Boston Dental College ........ 345
A Good Appliance ........ 346
At Last ........ 346
The Hygiene of the Mouth, a Guide to the Prevention and Control of Dental Diseases ........ 347

